# Exploring a Multi-Layer Coupled Network Propagation Model Based on Information Diffusion and Bounded Trust

**DOI:** 10.3389/ijph.2022.1604887

**Published:** 2022-07-18

**Authors:** Chunhua Ju, Chenyu Wang, Yihao Jiang, Fuguang Bao, Huajian Zhou, Chonghuan Xu

**Affiliations:** ^1^ School of Management Engineering and E-Commerce, Zhejiang Gongshang University, Hangzhou, China; ^2^ School of Business Administration, Zhejiang Gongshang University, Hangzhou, China

**Keywords:** rumor spreading, variation, coupled network, oyster, opinion interaction

## Abstract

**Objective:** To explore the law of opinion dissemination and individual opinion evolution at the micro level, this paper analyzes the influence of variation and oyster on communication from the perspective of network structure.

**Methods:** In this paper, we introduce the concepts of “variation” and “oyster”, build a multi-layer coupled network environment combined with the ISOVR model, and conduct simulation experiments of network information dissemination based on the bounded trust model.

**Results:** The experimental results reveal that the extent and scope of variation’s spread in the network are more dependent on the trust of nodes themselves, and decreasing the trust of nodes significantly reduces the rate and peak value of variation. Changing the silence coefficient of variation does not effectively change the direction of rumor propagation, which indicates that rumor has a strong propagation ability after mutation.

**Conclusion:** The insights of this paper on the dissemination of public opinions include: 1) pay attention to people with high trust levels, such as opinion leaders; 2) clarify the misinformation in time to prevent further spread of rumors.

## Introduction

With the rapid development of mobile networks and various media, the speed and scope of information dissemination have been greatly improved. When some unexpected events occur, all kinds of randomly fabricated and maliciously distorted news are widely spread [1]. New requirements have been put forward on how to effectively guide and control public opinions by governments and other public network opinion organizations. This paper analyses the characteristics of rumor spreading, extending its background to the coupled network, including layers of the online Internet, such as the intermediate transition layer and offline entity layer, and proposes a multi-layer coupled network based on information diffusion and bounded trust. The purpose is to obtain the rules of rumor spreading through different topological structures, to help the government control rumor spreading, and guide public opinions.

In terms of news dissemination, social media has increased the likelihood of one’s exposure to news events [2], also making the News Find Me effect more pronounced. However, the News Find Me effect causes people to receive information in a way that favors passive reception [3] and has no positive effect on the proper understanding of information [4]. Research on rumor spreading based on the infectious disease model began in the 1970s [5–10]. With further research, researchers have verified the fact that a larger trust parameter, forgetting rate, and time delay affect the speed of rumor spreading [11–13]. Du et al. [14] concluded that public education and timely correction of media errors were crucial factors of propagation. From the perspective of the heterogeneity of public risk perception, the lower the heterogeneity of public risk perception, the greater the impact on public opinions [15].

Researchers have used the viewpoint dynamics model to describe rumor propagation and changes in perspectives. Zha et al. [16] constructed a consensus-reaching model with a personalized feedback mechanism to propose acceptable opinions when the bounded confidence level is either known or unknown. Xiao et al. [17] verified that changes in individual opinions were not only influenced by interactions between adjacent nodes, but also by the natural renewal of individuals. Based on previous studies, Vasca et al. [18] constructed a bounded trust HK model with time heterogeneity to describe changes in group perspectives. Zhu et al. [19] divided people into opinion leaders and followers, and found that opinion leaders played a vital role in opinion convergence and initial opinion value. Then they added a trust incentive mechanism for uncorrected information and false information [20] and finally found that it was the copycat phenomenon that contributed to false advertising activities. Ju et al. [21] studied social relationship prediction by integrating personality traits and asymmetric interactions in a social network. The simulation experiment found that the discrete behavior would attract agents who trusted it and made them express extreme views.

More and more researchers have introduced the double-layer or multi-layer coupled network rumor spreading model. Zhang et al. [22] divided nodes into high influence layer and low influence layer, and designed two strategies of information closure and information elimination in rumor spreading. Barnard et al. [23] analyzed the evolution of infectious diseases by combining the association between individuals in the double static-dynamic layer network with time, type, and structural heterogeneity. Wang et al. [24] analyzed the impact of the social reinforcement effect, users’ perceived value, and other factors on rumor spreading from the perspective of user asymmetry in the network environment. Liu et al. [25] analyzed the local stability of the disease-free equilibrium point of the system according to the transformation law between different states in the model. Chen [26] constructed a multi-layer (WeChat layer-Weibo layer-control layer) coupled network model of public opinion communication control. Through simulation experiments, Zhu et al. [27] found that knowledge spreads fastest in the scale-free homogeneous hybrid double-layer coupled network. The communication frequency between online and offline knowledge was relatively close, the spread range was larger and the spread speed was faster.

In recent years, with deepening research on rumor propagation, scholars have analyzed the rules of rumor propagation from different perspectives [28–30], resulting in categories for the types and nature of rumor propagation, dissemination groups, network structure, and the polarization degree of individual opinions, etc. However, few studies have examined the mechanism of rumor spreading online and offline. Thus, this paper constructs a three-layer coupled network. The main contributions of this paper are as follows.

(1) An improved multi-layer coupled network structure is proposed to analyze the interaction of nodes both online and offline.
(2) The phenomena of “variation” and “oyster” are introduced and innovatively described from the perspective of viewpoint interaction.
(3) Experiments on multi-layer coupled network communication incorporating information diffusion and bounded trust are carried out, and the evolution of public opinion dissemination is analyzed from both macro and micro perspectives.

## Methods

**FIGURE 1:**
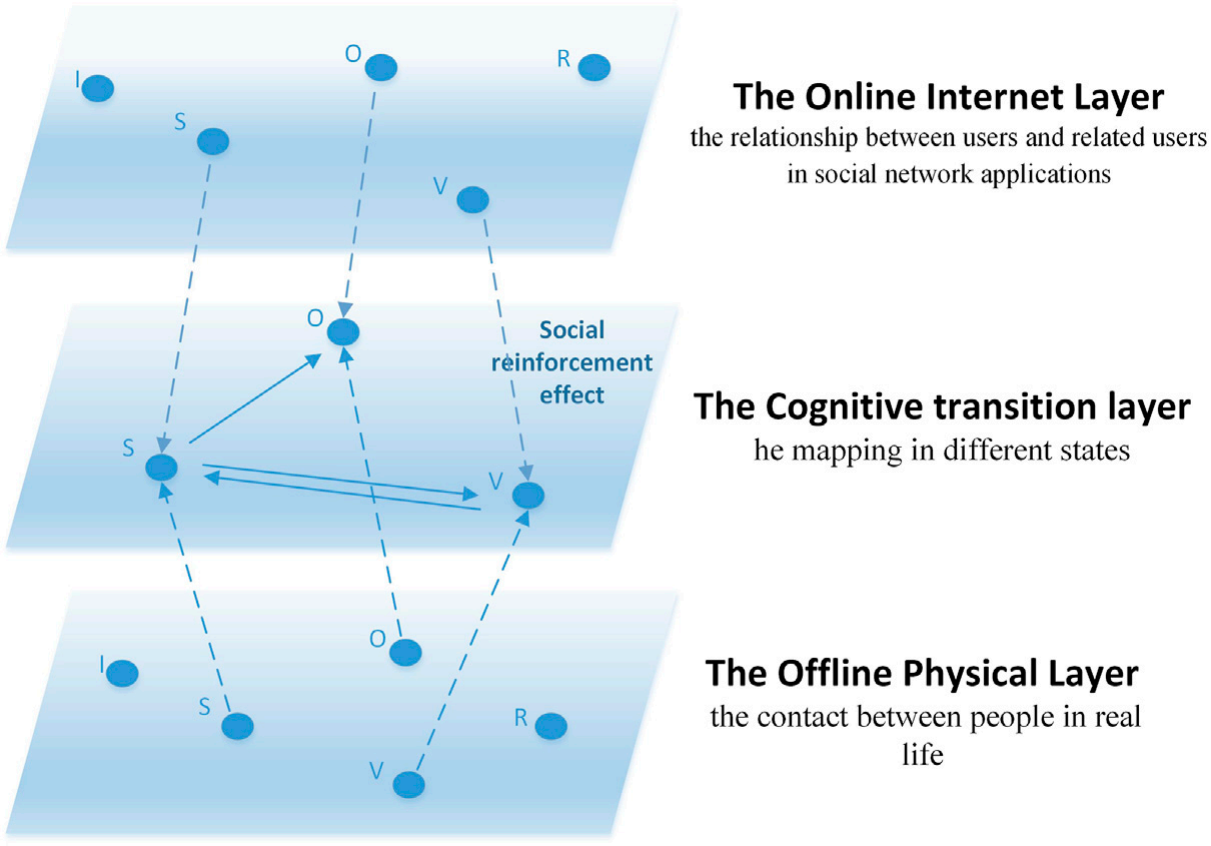
Three-layer coupled network with random online and offline coupling (Public opinion research, China, 2021-2022).

The main work of this paper is as follows: Firstly, referring to Ju et al. [31], we propose an ISVOR model with concepts of “variation” and “oyster”. According to the model, we divide people into three groups: I (Ignorant), S (Spreader), and V (Variation). Secondly, considering the situation of online and offline interactions in the social network era, a multi-layer coupled network of online Internet layer, intermediate transition layer, and offline entity layer is conducted. Thirdly, based on the model of online rumor spreading with variation and oyster phenomenon, information diffusion and bounded trust are introduced to explore distorted dissemination of information and the short period of stopping dissemination in the life cycle of a rumor. Figure 1 is a three-layer coupled network with random online and offline coupling.

For the online Internet layer and offline entity layer, the improved Amend-Linear Threshold (A-LT) model is used to represent the diffusion and elimination of rumors, and the J-A bounded trust model is used to describe the interactions between individuals. The intermediate transition layer of the three-layer coupled network in this paper adopts the random synchronization of the inter-layer state with the social reinforcement effect. The structure of the three-layer coupled network will be described in detail in *Three-Layer Coupled Network Structure* section.

### Characteristics of Individual Influence

The heterogeneity attribute of the node itself can promote or inhibit the spread of rumors [32]. This paper mainly considers the interaction between nodes and adjacent nodes. We use Degree Centrality and Eigen Centrality to describe the influence of structural dimensions of nodes in the propagation. And we set $a_{ij}=1$ to describe that a node  i  in a network is connected to  j .

1) Degree Centrality. The greater the degree of a node is, the greater the influence of this point will be. We use the following equation [33] to describe the degree of centrality of node i :

$$DE\left(i\right)=\frac{1}{n-1}\sum_{j=1}^{n}a_{ij}\;$$ (1)

2) Eigen Centrality. And the influence of nodes is distributed to all nodes in the network so that nodes with high centrality contribute more [34]. We use $x_{i}$ to indicate the level of importance by the following equations:

$$EC\left(i\right)=x_{i}=C\sum_{j=1}^{n}a_{ij}x_{j}\;$$ (2)

where C is a constant, $x={\left[x_{1},x_{2},x_{3},\ldots ,x_{n}\right]}^{T}$ . We can use the following equation to describe the steady state:

x=cAx (3)

where x is the eigenvector corresponding to the eigen values of matrix A .

3) Assuming that Ar(i) represents the attribute influence of different characters, *S, V, O,* and *R* represent the set of the spreader, the variation, the oyster, and the recovery.

$$Ar\left(i\right)=\{\begin{array}{cc} \alpha_{1} & i\in S\\ \alpha_{2} & i\in V\\ \alpha_{3} & i\in O \end{array}$$ (4)

This paper uses the structural dimension influence and attribute influence to determine the individual influence of node i in Internet Topology, denoted as influence(i) , and represented by the following formula:

influence(i)=DE(i)∗EC(i)∗Ar(i) (5)

In the online Internet layer, the probability of the node  i  being offline is denoted as ${\text{ o}}_{i}$ . When ${\text{ o}}_{i}=0.9$ , the node i has a 90% probability of being offline, meaning it is far away from rumor spreading in the network. Furthermore, it is not affected by other nodes in the network layer and does not participate in the information transmission.

### Activation and Extinction of Rumor Diffusion

In the improved linear threshold (A-LT) model, the nodes in the network are divided into three categories: ignorant nodes (I) that are not exposed to rumors or infected by rumors, carriers (C) that are infected with rumors and have the ability to spread rumors, and recoveries (R) that no longer spread rumors. This paper assumes that when the node energy received by the unknown node reaches the wake-up threshold, the node will be activated and become a carrier and spread the rumor to neighboring nodes. At the same time, when the energy of adjacent nodes of the activated carrier is lower than a certain value, the impact of rumors begins to disappear and the node state changes into an immune state. The value here is defined as the Extinguishment threshold.

**FIGURE 2:**
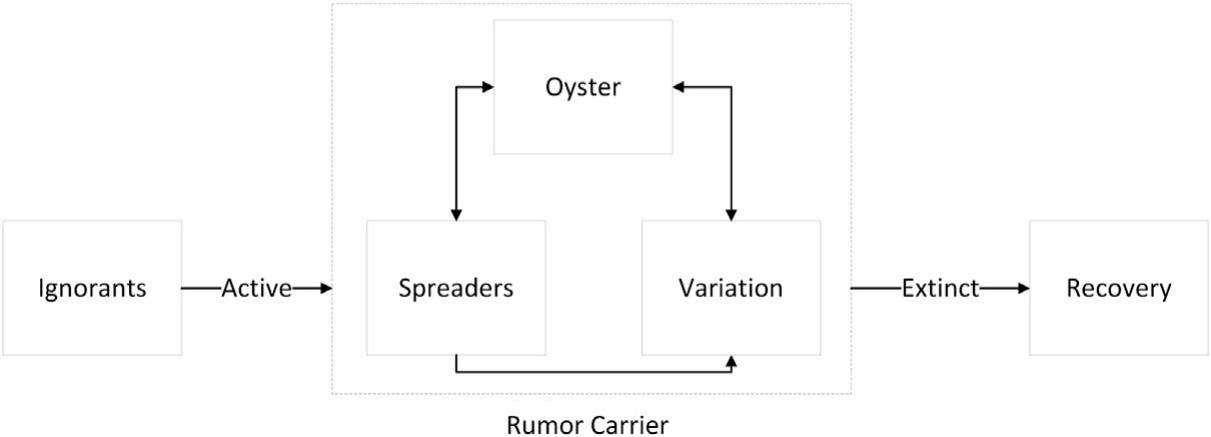
Activation and extinction process of rumor diffusion (Public opinion research, China, 2021-2022).

In the micro process of diffusion, rumor carriers can be subdivided into the spreader (S), variation (V), and the oyster (O), and the three groups of individuals have different influences. After a period of silence, some oysters will become immune to the rumor through rational thinking, and some of them will turn to the state of “spreading rumor”. They ignore the variation of rumors in the propagation and the influence of unstable factors of variation on the transmission chain. The state changes of users in the online Internet layer and offline entity layer are shown in Figure 2.

The total number of users in each layer is set as N , and at a certain time t , the relative number of N in different states during the activation of rumors, respectively represents the density of the ignorant I(t) , rumor carriers C(t) , spreaders S(t) , variations V(t) , the oyster O(t) , and the recovery R(t) . They all satisfy I(t)+C(t)+R(t)=1 , S(t)+V(t)+O(t)=C(t) .

In this model, the arousal threshold ϕ describes that the ignorant user adjusts the rumor acceptance level in combination with the surrounding environment and self-state. The larger the ϕ of node i , the less likely the rumor intrusion will be. Therefore, it can be assumed that the probability of an ignorant person in the network turning into the rumor-spreading state at time t+1 is as follows:

$$\theta \left(t+1\right)=\{\begin{array}{cc} 1 & \frac{Amount\left(C\right)}{Amount\left(All\right)}\geq \phi \\ 0 & \frac{Amount\left(C\right)}{Amount\left(All\right)}<\phi \end{array}$$ (6)

where Amount(C) represents the sum of the number of all rumor carriers in the adjacent nodes of node i at time t , and  Amount(All) represents the sum of all rumor carriers in adjacent nodes of node i . Without loss of generality, we assume that the wake-up threshold ϕ of all users obeys a general distribution, denoting $\phi_{i}∼N\left(\bar{\phi },\sigma_{1}\right)$ .

This model uses the blanking threshold φ . The larger the φ  of node i , the better it can distinguish right from wrong, and the easier it is to identify rumors. We assume that the probability of the ignorant transforming into the recovery in the network at time t+1 is as follows:

$$\vartheta \left(t+1\right)=\{\begin{array}{cc} 1 & \frac{Amount\left(C\right)}{Amount\left(All\right)}\geq \varphi \\ 0 & \frac{Amount\left(C\right)}{Amount\left(All\right)}<\varphi \end{array}$$ (7)

We assume that the extinguishing threshold φ of all users obeys a normal distribution, denoting $\varphi_{i}∼N\left(\bar{\varphi },\sigma_{2}\right)$ .

We use influence(All) to represent the sum of the influences of all activated nodes in the adjacent nodes of the node i . The influence(S) represents the sum of the influences of all ordinary spreaders. The influence(V) represents the sum of the influences of the change. The influence(O) represents the sum of the influences of the oyster. A random constant r is generated between [0, 1] , and the state $x_{t+1}$ of node  i at the next moment is expressed by the following formula:

$$x_{t+1}=\{\begin{array}{cc} S & 0\leq r<\frac{influence\left(S\right)}{influence\left(All\right)}\\ V & \frac{influence\left(S\right)}{influence\left(All\right)}\leq r<\frac{influence\left(S+V\right)}{influence\left(All\right)}\\ O & \frac{influence\left(S+V\right)}{influence\left(All\right)}\leq r<\frac{influence\left(All\right)}{influence\left(All\right)} \end{array}$$ (8)

### Variation and Oyster in Rumor Spreading

There will be the phenomenon of silence and variation in group communication. We call these people oysters and variations. Different from ordinary rumor spreaders, the content spread by Variations has changed significantly with the change in how news and specific information are disseminated, and there is a tendency for constant change. Oysters receive rumors but do not take the initiative to spread rumors. They are either in a thinking state or adopt a wait-and-see attitude.

We assume that the initial value $x_{i}$ of the node that has just entered the propagation state is in [−1,+1] , is subject to the random distribution. We stipulate that when the opinion value is in $\left[0,\rho_{3}\right]$ or $\left[\rho_{4},1\right]$ , it means that the node is in the state of ordinary spreader; when the opinion value is in $\left[-1,\rho_{1}\right]$ or $\left[-\rho_{2},0\right]$ , it means that the node is in the state of variation; when the opinion value is in $\left[\rho_{1},\rho_{2}\right]$ and $\left[\rho_{3},\rho_{4}\right]$ , it means that the node is an oyster at the moment. Among them, $\;\left[\rho_{1},\rho_{2}\right]$ and $\left[\rho_{3},\rho_{4}\right]$ are the silence intervals between the variation and the spreader, and $\rho_{1}$ and $\rho_{2}$ , $\rho_{3}$ and $\rho_{4}$ are the silent coefficients of the variation and the spreader.

Referring to the JA model of Jager et al. [35], the rules of opinion update are as follows: when the difference between two opinion values is within the allowed interval $d_{1}$ , mutual influences and the change of opinion values will occur, which is expressed by the following equations:

$$x_{i}\left(t+1\right)=x_{i}\left(t\right)+\mu \left|x_{i}\left(t\right)-X_{j}\left(t\right)\right|\left|x_{i}\left(t\right)-X_{j}\left(t\right)\right|<d_{1}$$ (9)

where the $X_{j}\left(t\right)$ represents the average value of the sum of opinion values around the node.

When the difference of opinion values is out of the interval $d_{2}$ , it means that the opinions among nodes are different and inconsistent and there will be an exclusion between nodes, meaning the difference of opinion values will further expand, as shown in the following formula:

$$x_{i}\left(t+1\right)=x_{i}\left(t\right)-\mu \left|x_{i}\left(t\right)-X_{j}\left(t\right)\right|\left|x_{i}\left(t\right)-X_{i}\left(t\right)\right|>d_{2}$$ (10)

When the opinion values of other intervals are different, there is no willingness to communicate between nodes, so the opinion values of nodes will not change. Meanwhile, we stipulate that once the opinion value interval of nodes is in $\left[0,\rho_{3}\right]$ , the opinion value will not reduce in the interaction progress, nor will it be reversed to an ordinary communicator.

### Three-Layer Coupled Network Structure

In the intermediate cognitive transition layer, the social reinforcement effect is redefined as [36]: nodes in the network layer are influenced by multiple neighboring nodes. The probability of random synchronization from the intermediate cognitive transition layer to the offline entity layer in the online Internet layer [37] is:

$$\bar{\omega }\left(n\right)=\frac{\xi n}{1+\gamma n}$$ (11)

where ξ represents the random state synchronization parameter, and n represents the number of connected users in the same type. β represents the social reinforcement parameter, which represents external factors received by the node, including the influence of regulators (such as the media). We set 0<γ<1 . When the generated random number satisfies the random synchronization probability of social reinforcement, the state of the online internet layer will be synchronized to the offline state.

The connection mode of nodes at different layers adopts the combination of the following three modes [38, 39]: 1) assortative links between layers; 2) disassortative links between layers, which is the opposite of assortative links between layers; and 3) random links between layers.

**FIGURE 3:**
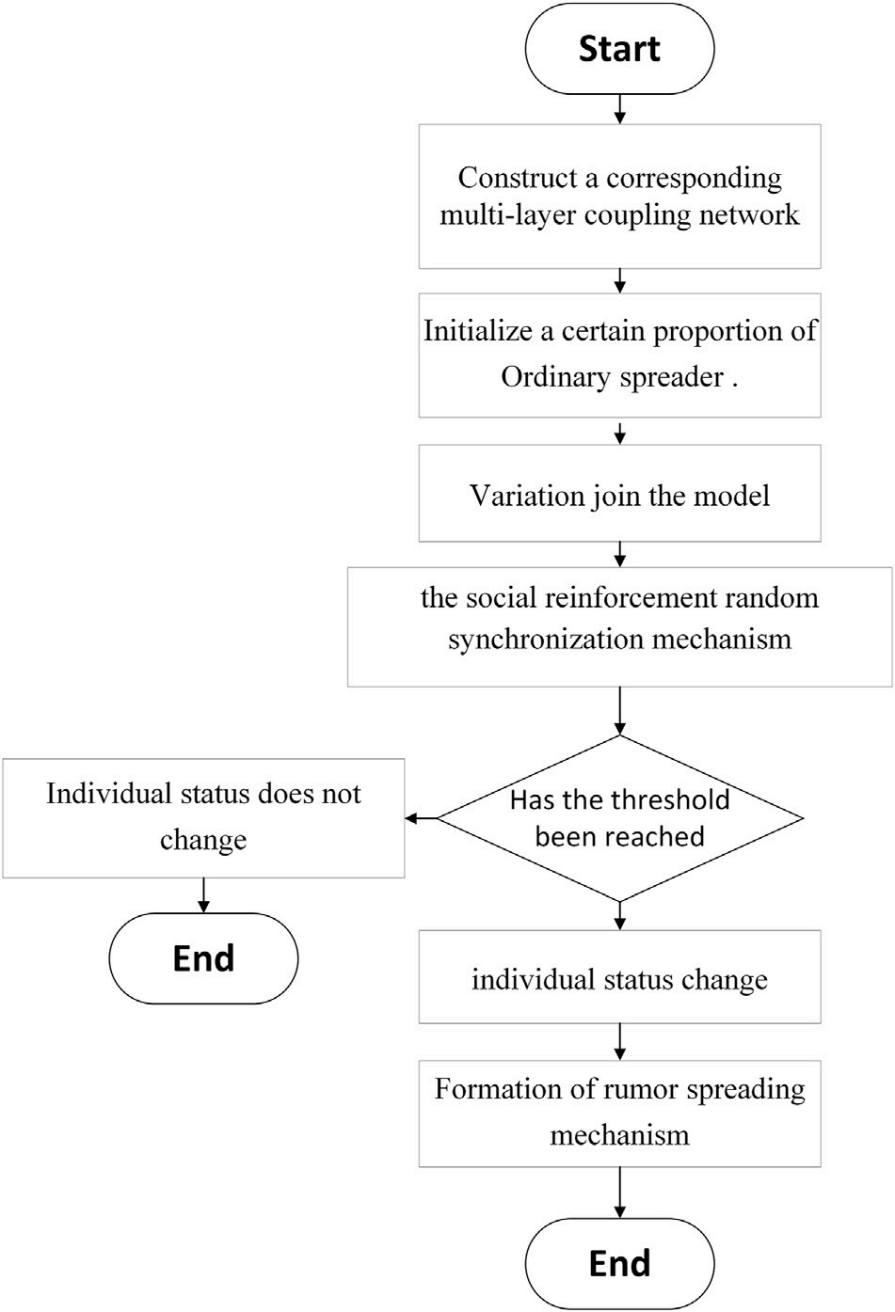
Experimental flow chart (Public opinion research, China, 2021-2022).

For the topological structure of social networks, the activation and elimination of rumor diffusion, and allowing for the variations and oysters in the process, this paper makes the following provisions: 1) the topology of all networks is static; 2) there is no after-effects in the online internet layer and the offline entity layer; 3) the node relationship between the online internet layer and the offline entity layer is bidirectional; and 4) the nodes between the online Internet layer and the offline entity layer are one-on-one. Figure 3 is the experimental flow chart of this paper.

## Result

In this section, we use the Monte Carlo method to simulate on the Python platform. In order to verify the scientificity and reliability of the above model, this paper selected the “Liu Xuezhou Incident”, which was fermented in the campus forum as a case study, verified the science of the three-layer coupled network communication model based on case data, and conducted simulation experiments.

**FIGURE 4:**
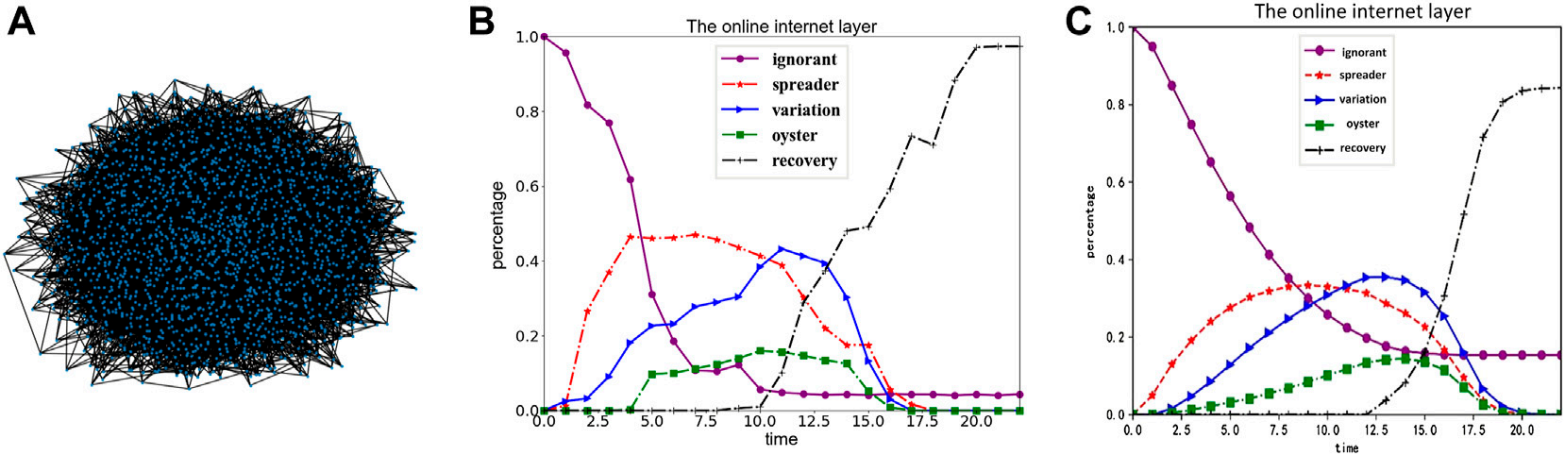
Comparison of real data and simulation data (Public opinion research, China, 2021-2022). **(A)** shows the user interaction network diagram presented according to the actual campus forum user; **(B)** shows the trend graph of the population change obtained according to the proposed user data and model; **(C)** shows the population change trend graph obtained according to the parameters mentioned in the text.

Brief description of the incident: “Liu Xuezhou Incident” was a public opinion event that occurred at the end of 2021. The origin of the incident is as follows: On 6 December 2021, Liu Xuezhou posted a video of a family search on the Internet, which aroused the attention of the general public. On 16 December, the elderly members of the family found Liu Xuezhou’s biological parents through the vaccine book of that year, and the two sides recognized each other through DNA comparison. However, on 17 January 2022, Liu Xuezhou posted an article on a social media platform claiming that they were abandoned by their biological parents. The dispute started because they had hoped that their biological parents could provide a place for them to live. The media also published one-sided and suggestive headline articles to mislead netizens. In the early morning of 24 January, Liu Xuezhou committed suicide after posting a long article. This paper used the content of all users in the #LiuXueZhou incident topic from 6 December 2021, to 31 January 2022, which resulted in 2078 user nodes after filtering out irrelevant data. We defined user interactions in terms of re-Tweets (on the social media platform Twitter) and comments on published content. We finally achieved the following user interaction network diagram based on the interaction between users, shown in Figure 4A. Meanwhile, based on the user data we trawled, the trend graph of the population was obtained through the calculation of the above model, as shown in Figure 4B.

According to the Figures 4A,B, this topic can be abstracted as a BA scale-free network, which we simplified to a scale-free network with N=2000, m=5, <k>=10 to facilitate subsequent calculations. Based on the above data, the paper sets each initial parameter as follows. The offline probability of the online internet layer is  ο=0.97 . The average value of the rumor awakening threshold is $\bar{\phi }=0.2$ , namely variance $\sigma_{1}=0.5$ . The average value of the rumor elimination threshold is $\bar{\varphi }=0.2$ , namely variance $\sigma_{2}=0.15$ . The random synchronization parameter of social reinforcement is  ξ=0.1 , and the social reinforcement factor is  η=0.2 . The attribute influences of the spreader, the variation, and the oyster are $Ar_{S}=1$ , $\;Ar_{V}=1.5$ , $\;Ar_{O}=0.5$ , respectively. The immune appears at t=15 . The individual’s bounded trust degree is   μ=0.1 , and the assimilation distance of the bounded trust view is $d_{1}=1$ , and the exclusion distance is $d_{2}=1.5$ . The silence coefficients of spreaders and variations are $\;\rho_{1}$ = -0.4, $\rho_{2}$ = −0.2, $\rho_{3}$ = 0.2 and $\rho_{4}$ = 0.3. In the case of the above parameters, we plotted the trend of population change, as shown in Figure 4C. Through comparative analysis, the population change trend obtained by the above parameters is consistent with the population change trend in the actual situation. Therefore, the three-layer coupled network structure proposed in this paper is consistent with the actual situation, and the above parameter settings are used in the subsequent simulation experiments of this paper.

**FIGURE 5:**
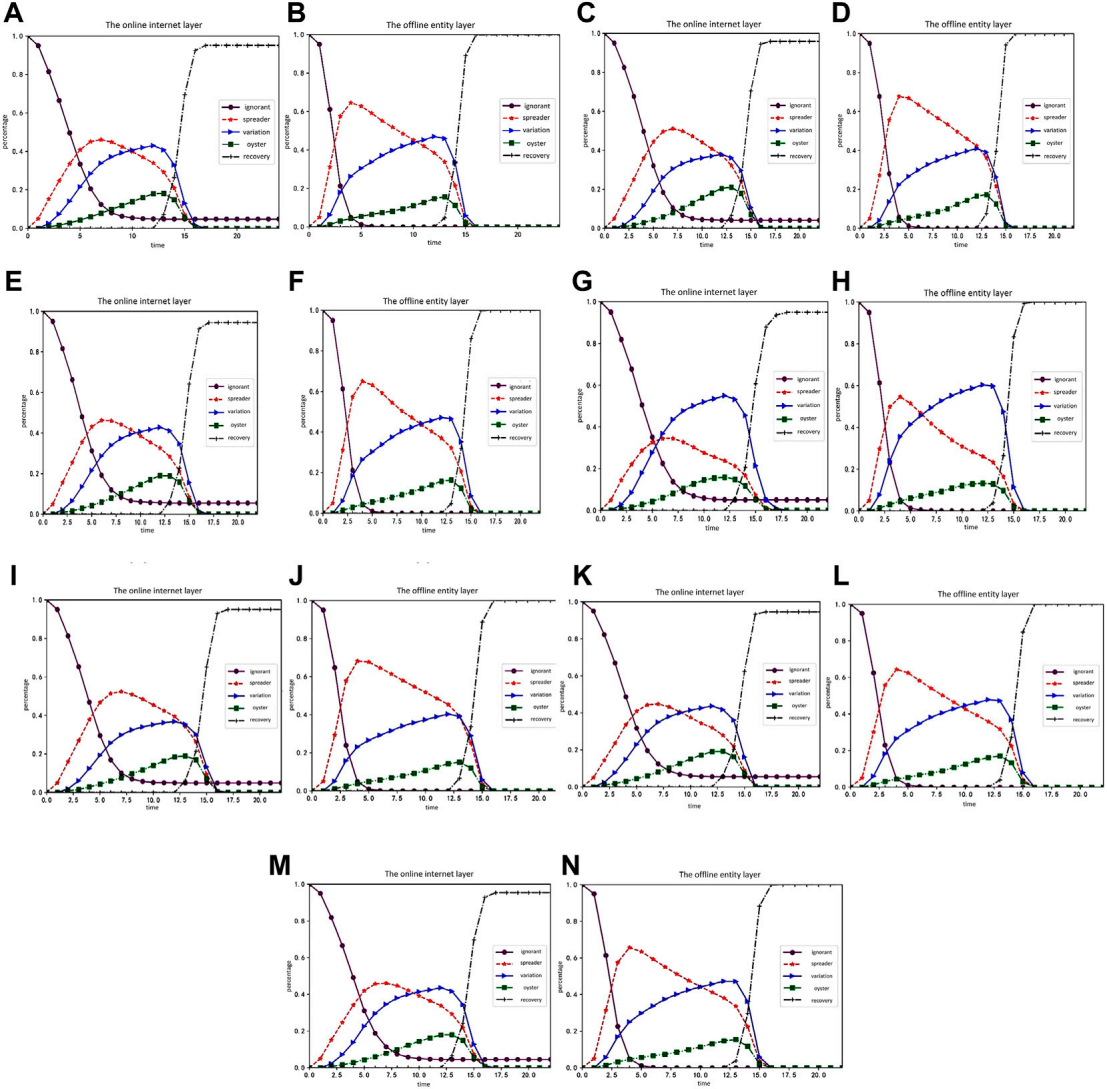
Simulation experiment result graph 1 (Public opinion research, China, 2021-2022). **(A,B)** show the time-varying curves of the proportion of the ignorant, communicator, mutants, oysters, and the immunity in the online internet layer and offline physical layer. **(C–H)** shows changes in the proportion of each character in the online internet layer and offline entity layer over time when the attribute influences of the mutant ArV are 1.5, 2.0, and 2.5, respectively. **(I–N)** shows the change of the proportion of each character in the online internet layer and offline entity layer over time when the attribute influences of oysters ArO are 0.25, 0.5, and 0.75, respectively.

**FIGURE 6:**
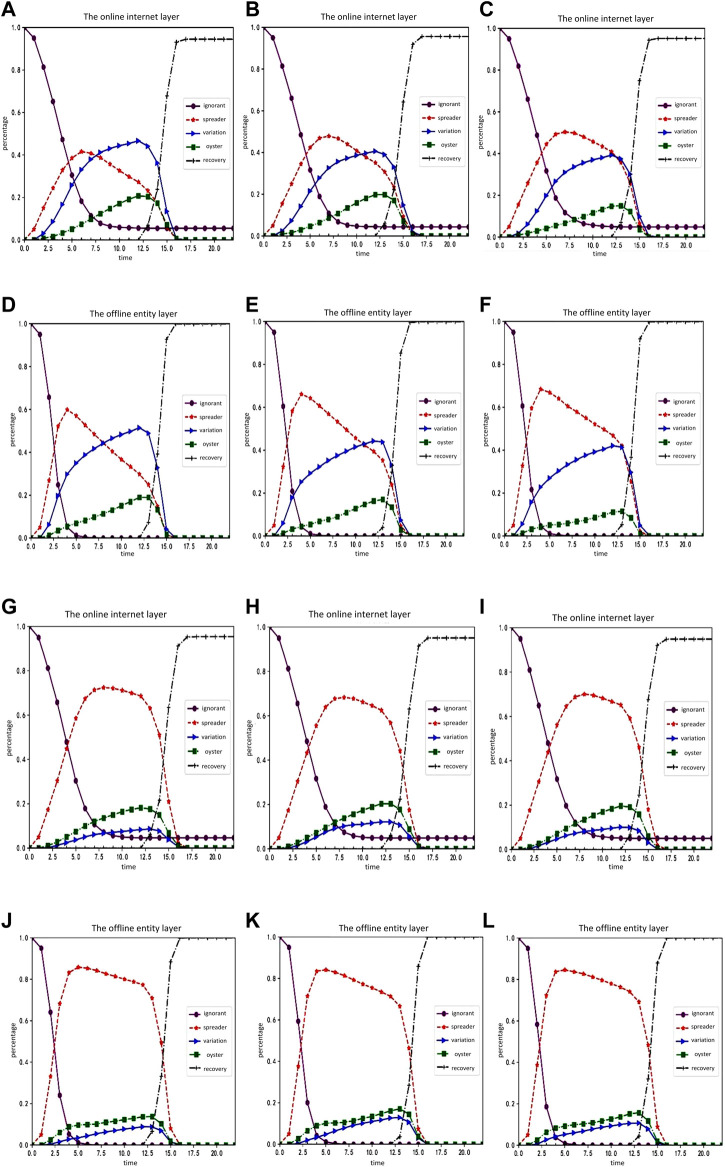
Simulation experiment result graph 2 (Public opinion research, China, 2021-2022). **(A–F)** shows changes in the proportion of each character in the online internet layer and offline entity layer over time when the communicator's silence coefficient ρ1 is −0.45, −0.4 and −0.35, respectively. **(G–L)** shows the changes of the proportion of each character in the online internet layer and offline entity layer over time when the variation’s silent coefficient ρ3 is 0.15, 0.2 and 0.25, respectively.

The order of the experiment is the change of the proportion of each character, followed by the change of the random social reinforcement effect. We then examined the influence of variation and oyster attributes, the change of trust degree of nodes, and the change and variation of communicator silence coefficient. The rule of rumor spreading is analyzed through the parameter changes of influencing factors. The experimental results are shown in Figures 5, 6:

Figures 5A,B shows the time-varying curves of the proportion of the ignorant, communicator, mutants, oysters, and the immunity in the online internet layer and offline physical layer. Figures 5C–H shows changes in the proportion of each character in the online internet layer and offline entity layer over time when the attribute influences of the mutant $Ar_{V}$ are 1.5, 2.0, and 2.5, respectively. Figures 5I–N shows the change of the proportion of each character in the online internet layer and offline entity layer over time when the attribute influences of oysters $Ar_{O}$ are 0.25, 0.5, and 0.75, respectively.

Figures 6A–F shows changes in the proportion of each character in the online internet layer and offline entity layer over time when the communicator’s silence coefficient $\rho_{1}$ is −0.45, −0.4 and −0.35, respectively. Figures 6G–L shows the changes of the proportion of each character in the online internet layer and offline entity layer over time when the variation’s silent coefficient $\rho_{3}$ is 0.15, 0.2 and 0.25, respectively.

Based on a comprehensive comparative analysis, the following conclusion can be drawn: 1) each character in the offline physical layer changes in the same way as that in the online internet layer; 2) compared with the situation without random social reinforcement, the ignorant people in the offline physical layer are more likely to be infected by rumors and keep up with online events due to the superimposed influence of rumor carriers in the online internet layer; 3) when the attribute influence of mutants increases, the growth rate of silent mutants decreases, and the max{O(t)} of silent mutants also drops slightly, but it is not significantly affected; 4) with the increase of the attribute influence of the silent, the proportion of communicators in the online internet layer has no obvious change, while the proportion of mutants is constantly increasing; 5) with the increase of the degree of bounded trust, the proportion of communicators in the online internet layer and the offline physical layer grows flat, while the proportion of mutants grows faster and the proportion of silent ones increases as well. Finally, the max{O(t)} decreases slightly, but it is not significantly affected; 6) changing the silence coefficient of communicators is less effective than changing the silence coefficient of mutants.

## Discussion

In this paper, we draw on an ISOVR model of Jiang et al. [31] and construct a multi-coupled network to analyze the online and offline spread of rumors. Meanwhile, we introduce the bounded trust model and social reinforcement effect for further study. The simulation results show that increasing the immunity rate and silence rate of the variation can effectively reduce the rate of rumor diffusion. The high random social reinforcement effect will promote the spread of rumors in the offline entity layer. The offline probability of nodes within a certain range will effectively control the spread of rumors in the online internet layer. In addition, the degree and scope of variation diffusion in the network depend more on the trust degree of the node itself. Increasing the trust degree of nodes can significantly promote the growth rate and peak value of the variation. Therefore, it is necessary to pay attention to the cultivation of influential groups that can play a positive role in the spread of rumors. Changing the silence coefficient of variation cannot control the rumor diffusion, which shows the powerful influence of rumor variation. Therefore, relevant departments should release true information in a timely way to eliminate rumors and prevent further variation and distortion of rumors.

### Conclusion

This paper studies the variations and oysters in network communication and puts forward a new transmission mechanism and introduces the phenomenon of stagnation and information mutation in the network. The model has a wide range of application scenarios in the future, including personalized recommendation [40–43], data processing [44–46], sustainable tourism [47, 48], knowledge dissemination [49] and so on. At the same time, there are still some limitations, for example, although the parameters of this paper were obtained after the simulation of actual cases, it is not considered in the later analysis that the parameters will change dynamically according to the development of actual events [50]. As mentioned in this study, it is necessary to strengthen the cultivation of groups that have a positive impact on the spread of rumors. However, this paper does not cover how to identify and cultivate such groups, nor how to guide the dissemination and control of public opinion based on the feedback between groups [51]. In follow-up research, we will further analyze the influence of increasing intervention measures and inter-group feedback on the dissemination of false mutation information in the process of public opinion dissemination. We might also consider combining the optimal control model, taking the control cost of public opinion dissemination as the objective function to dynamically adjust the impact of the intervention strategy on information dissemination.
